# Data on uncoupling protein-3 levels, hypoxia, low flow ischemia, and insulin stimulation in dystrophin-deficient *mdx* mouse hearts

**DOI:** 10.1016/j.dib.2018.08.010

**Published:** 2018-08-08

**Authors:** Wen Zhang, A. Elizabeth Sang, Michiel ten Hove, Stefan Neubauer, Kieran Clarke

**Affiliations:** a Department of Physiology, Anatomy and Genetics, University of Oxford, Sherrington Building, Parks Road, Oxford OX1 3PT, United Kingdom; b Radcliffe Department of Medicine, University of Oxford, Oxford, United Kingdom

## Abstract

The data contain body weights, plasma free fatty acids concentrations and cardiac uncoupling protein-3 protein levels for wild-type and *mdx* mice. The data provide heart rates, left ventricular contractile functions, coronary flow, phosphocreatine concentrations, and adenosine 5’-triphosphate (ATP) concentrations throughout hypoxia in *mdx* mouse hearts. This data article also provides left ventricular contractile functions after low flow ischemia with and without glucose, glycogen levels before ischemia or hypoxia, glucose uptake rates during low flow ischemia and insulin stimulation, and insulin-stimulated phospho-Akt protein levels, a protein in insulin signaling, in *mdx* mouse hearts.

## Specifications Table

**Table:** 

Subject area	Medical sciences
More specific subject area	Cardiac magnetic resonance spectroscopy and signaling pathways
Type of data	Figure
How data was acquired	Magnetic resonance spectroscopy, hypoxia and low flow ischaemia using isolated whole Langendorff-perfused mouse hearts
Data format	Analyzed
Experimental factors	Male mice were used.
Experimental features	The effects of dystrophin deficiency on uncoupling protein-3 levels, glycogen levels, phosphocreatine and ATP concentrations throughout hypoxia, glucose uptake rates during low flow ischemia and insulin stimulation using D-[2–^3^H] glucose in *mdx* mouse hearts were determined.
Data source location	University of Oxford, United Kingdom
Data accessibility	All data are available with this article.

## Value of the data

• This data can be used to further investigate the role of dystrophin in obesity and elevated serum free fatty acids levels in human Duchenne muscular dystrophy patients.
• The data can be used to determine whether decreased phosphocreatine and ATP concentrations cause decreased contractile functions after hypoxia or vice verse in *mdx* mouse hearts.
• Glucose uptake rates and phospho-Akt data can be used to investigate the role of dystrophin in signaling.

## 1 Data

*Mdx* mice have abnormal body weights, plasma free fatty acids concentrations and cardiac uncoupling protein-3 protein levels compared with wild-type mice. *Mdx* mouse hearts have decreased left ventricular contractile functions and faster ATP loss rates during hypoxia compared with wild-types. *Mdx* mouse hearts also have higher left ventricular end-diastolic pressures and lower glucose uptake rates during low flow ischemia than wild-types. Insulin-stimulated phospho-Akt protein levels are similar in all mouse hearts while *mdx* mouse hearts have lower glucose uptake rates in response to insulin than wild-types.

## 2 Experimental design, materials, and methods

**Fig. 1:**
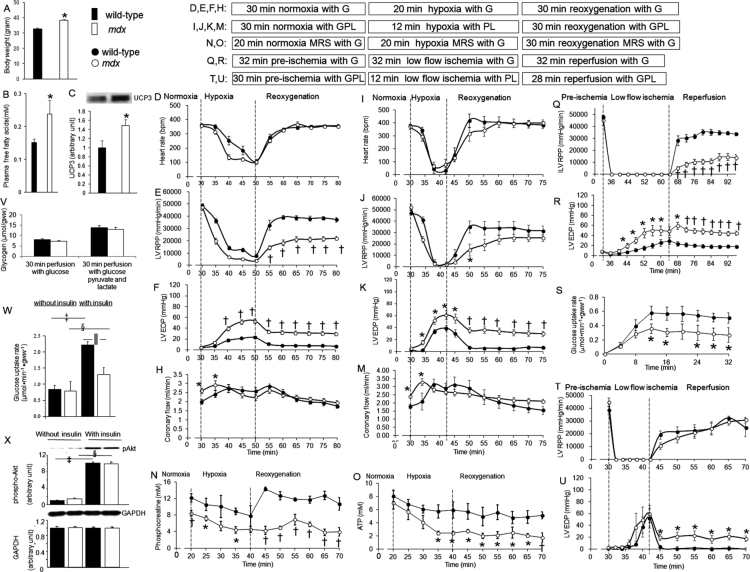
Body weights, plasma free fatty acids, western blot, hypoxia, low flow ischemia, and glycogen data. A: Body weights (*n* = 28 for wild-type, *n* = 30 for *mdx*); B: Plasma free fatty acids concentrations (*n* = 4 for wild-type, *n* = 5 for *mdx*); C: Uncoupling protein-3 (UCP3) western blot (*n* = 5 for wild-type, *n* = 5 for *mdx*); D–F, H: Hypoxia with glucose (*n* = 10 for wild-type; *n* = 12 for *mdx*); I–K, M: Hypoxia with pyruvate and lactate (*n* = 4 for wild-type; *n* = 5 for *mdx*); N, O: Phosphocreatine and ATP concentrations throughout hypoxia (*n* = 4 for wild-type, *n* = 5 for *mdx*). Q, R: Low flow ischemia with glucose (*n* = 10 for wild-type; *n* = 9 for *mdx*); S: Glucose uptake rates during low flow ischemia (*n* = 6 for wild-type; *n* = 5 for *mdx*); T, U: Low flow ischemia with pyruvate and lactate (*n* = 4 for wild-type; *n* = 5 for *mdx*); V: Cardiac glycogen levels (*n* = 6 for wild-type and *n* = 5 for *mdx* in both perfusions); W, X: Insulin-stimulated glucose uptake rates and phospho-Akt western blot (*n* = 5 for wild-type without and with insulin; *n* = 6 for *mdx* without and with insulin). LV RPP, left ventricular rate-pressure product; EDP, end-diastolic pressure; MRS, magnetic resonance spectroscopy. Substrate G: 11 mM glucose; GPL: 11 mM glucose, 1.8 mM pyruvate and 0.2 mM lactate; PL: 1.8 mM pyruvate and 0.2 mM lactate. Data are expressed as means ± SEM. * *P* < 0.05 vs. wild-type; † *P* < 0.01 vs. wild-type; ‡ *P* < 0.001 vs. non-insulin-stimulated wild-type; § *P* < 0.001 vs. non-insulin-stimulated *mdx*; ǁ *P* < 0.05 vs. insulin-stimulated wild-type.

Experiments were carried out using male wild-type (C57BL10) (*n* = 61) and age-matched *mdx* (*n* = 62) mice at 8 months. All the procedures were approved by the Animal Ethics Review Committees, University of Oxford, and by the Home Office, United Kingdom. Plasma free fatty acids concentrations were measured using NEFA kit (Wako Chemicals). Western blot quantified protein levels with anti-uncoupling protein-3, anti-phospho-Akt (Cell Signaling) and anti-GAPDH (Abcam). Isolated whole Langendorff-perfused mouse hearts were equilibrated with fatty acids free Krebs-Henseleit buffer gassed with 95% O_2_ – 5% CO_2_ (PaO_2_ = ~ 90 mmHg), and subjected to hypoxia with buffer gassed with 95% N_2_ – 5% CO_2_ (PaO_2_ = ~ 45 mmHg) and reoxygenation. Left ventricular pressures and coronary flow were recorded. Protocols were shown in Fig. 1. Glycogen levels before ischemia or hypoxia were determined [1]. Glucose uptake rates were calculated using D-[2–^3^H] glucose during low flow ischemia (0.5 ml min^−1^ gww^−1^) and in response to insulin [2]. Statistical significance was assessed using one-way analysis of variance followed by Student׳s *t*-test.
